# Strategies for the implementation of an electronic fracture risk assessment tool in long term care: a qualitative study

**DOI:** 10.1186/s12877-021-02388-3

**Published:** 2021-08-21

**Authors:** Yuxin Bai, Caitlin McArthur, George Ioannidis, Lora Giangregorio, Sharon Straus, Alexandra Papaioannou

**Affiliations:** 1 McMaster University, 1280 Main Street West, L8S 4L8 Hamilton, Ontario Canada; 2 GERAS Centre for Aging Research, 88 Maplewood Avenue, L8M 1W9 Hamilton, Ontario Canada; 3 Dalhousie University, PO Box 15000, 6299 South St, NS B3H 4R2 Halifax, Canada; 4 University of Waterloo, 200 University Avenue West, N2L 3G1 Waterloo, Ontario Canada; 5 Schlegel-UW Research Institute for Aging, 150 Laurelwood Drive, N2J 0E2 Waterloo, Ontario Canada; 6 University of Toronto, 27 King’s College Circle, M5S 1A4 Toronto, Ontario Canada

**Keywords:** Long-term care, Evidence-based, Guidelines, Knowledge translation, Fracture risk, Clinical assessment protocol

## Abstract

**Background:**

Older adults in long-term care (LTC) homes experience high rates of fractures, which are detrimental to their quality of life. The purpose of this study is to identify and make recommendations on strategies to implementing an evidence-based Fracture Risk Clinical Assessment Protocol (CAP) in LTC.

**Methods:**

Following the Behaviour Change Wheel framework, we conducted six focus group interviews with a total of 32 LTC stakeholders (e.g. LTC physicians) to identify barriers and facilitators, suggest implementation strategies, and discuss whether the identified strategies were affordable, practicable, effective, acceptable, safe, and if they promote equity (APEASE). The interviews were transcribed verbatim and analyzed using thematic content analysis.

**Results:**

Themes of implementation strategies that met the APEASE criteria were minimizing any increase in workload, training on CAP usage, education for residents and families, and persuasion through stories. Other strategy themes identified were culture change, resident-centred care, physical restructuring, software features, modeling in training, education for staff, social rewards, material rewards, public benchmarking, and regulations.

**Conclusions:**

To implement the Fracture Risk CAP in LTC, we recommend using implementation strategies centred around minimizing any increase in workload, training on CAP usage, providing education for residents and families, and persuading through stories. Through improving implementation of the fracture risk CAP, results from this work will improve identification and management of LTC residents at high fracture risk and could inform the implementation of guidelines for other conditions in LTC homes.

**Supplementary Information:**

The online version contains supplementary material available at 10.1186/s12877-021-02388-3.

## Introduction

Older adults experience significant deterioration in their health-related quality of life after a fracture, as well as changes in their capacity for mobility, self-care, and daily activities [1, 2]. Up to 85 % of long-term care (LTC) residents have osteoporosis, a skeletal disease characterized by bone deterioration and increased fracture risk [3]. Compared to age and sex-matched older adults in the community, LTC residents experience a higher fracture rate, lower likelihood of regaining their previous level of function, and higher chance of dying within 3 months post-fracture [4–7]. Despite the high incidence and severe consequences of fractures, guidelines for fracture risk assessment and management are underutilized in LTC [8, 9]. Furthermore, LTC-specific challenges make it difficult to use general fracture risk assessment tools. Indeed, while the median survival rate among residents were reported to be from 28 to 41 months [10], the Canadian Association of Radiologists and Osteoporosis Canada (CAROC) tool [11] and the Canadian WHO Fracture Risk Assessment Tool (FRAX) [12] estimate a 10-year fracture risk and thus cannot identify residents at imminent fracture risk (i.e. within the next year) for urgent prevention strategies. Additionally, cognitive impairment and frailty among LTC residents are not considered in CAROC or FRAX though those factors may contribute to fractures [13].

To address the barriers to fracture assessment and management [14], our team developed an evidence-based Fracture Risk Clinical Assessment Protocol (CAP) [15]. CAPs are decision-making tools that identify residents at risk for adverse outcomes and provide risk level-specific recommendations to clinicians [16]. CAPs are triggered by embedded risk algorithms in mandated LTC resident assessments that are stored as electronic medical records (RAI 2.0 and interRAI instrument) across Canada [17]. The Fracture Risk CAP is triggered by the embedded Fracture Risk Scale, an algorithm that identifies residents at high fracture risk by incorporating risk factors for osteoporosis and falls and delineates fracture prevention interventions [18]. The suggested prevention interventions are based on LTC-specific fracture prevention recommendations that were developed using the Grading of Recommendations Assessment, Development and Evaluation (GRADE) approach, which is a widely adopted framework for grading the quality of evidence and making clinical practice recommendations [19, 20]. Our team has developed the Fracture Risk CAP, and the next step is to determine the best way to support implementation to change practice.

Developing evidence-based guidelines and tools (e.g., the Fracture Risk CAP) is an early step in sustainable practice change [21, 22]. Indeed, failure to implement well-validated clinical interventions (including guidelines and tools) is a widespread problem [23]. Therefore, there is an urgent need for finding the best ways to implement guidelines and tools in LTC [24]. Due to the ineffectiveness of trial-and-error approaches to evidence uptake, implementation scientists call for theory-informed approaches to knowledge translation, defined as methods for closing the gaps from knowledge to practice [25, 26]. Throughout this manuscript, we will call these methods implementation strategies, defined as actions taken to enhance adoption, implementation, and sustainability of clinical interventions which when combined, forms an implementation intervention [27, 28].

The Behaviour Change Wheel (BCW) is an evidence-based framework to guide intervention design and implementation [29]. The guide is divided into three main stages: (1) Understand the behaviour, (2) Identify intervention options, and (3) Identify content and implementation options [30]. The BCW invites researchers to first identify the sources of behaviour (e.g. barriers and facilitators) in terms of COM-B components (Capability, Opportunity, Motivation are needed to change Behaviour) [30]. Next, it connects the COM-B components to nine intervention functions (education, training, environmental restructuring, restriction, coercion, incentivization, persuasion, modelling, enablement) that could change each of the COM-B components to overcome barriers and leverage facilitators [30]. Finally, the BCW encourages researchers to consider modes of delivery and select specific interventions using the APEASE criteria, which asks whether the intervention is Affordable, Practicable, Effective, Acceptable, Safe, and promotes Equity [30]. The BCW constructs and APEASE are defined in Additional File 1.

Guided by the BCW, the aims of the current study are to (1) identify implementation strategies based on barriers and facilitators to implementation in LTC; and (2) to make recommendations on how to implement the Fracture Risk CAP.

## Methods

**Fig. 1:**
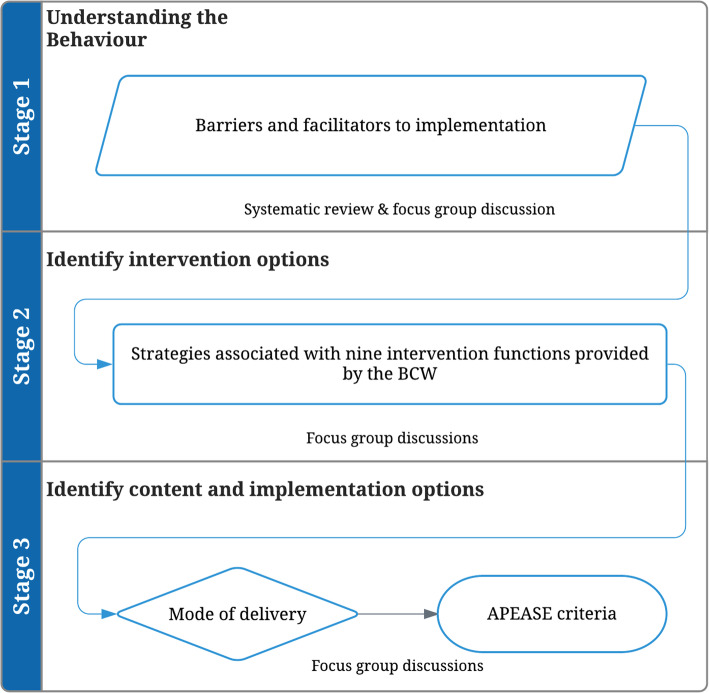
Flowchart of methods informed by the BCW

Our study was guided by the three stages of the BCW (Fig. 1).

### Setting and participants

We aimed to recruit a multidisciplinary group of stakeholders with expertise in fracture prevention in LTC from across Canada. We invited by email experts in LTC and fracture prevention to participate in a national face-to-face meeting to discuss preventing fractures in LTC. The health professionals were chosen based on their experience working in LTC or their expertise in fracture prevention. LTC residents and family members who were invited had prior experiences with fractures. All participants were recruited through word of mouth or were known to the team. Participants included physicians (e.g. family physicians and geriatricians), pharmacists, physiotherapists, kinesiologists, nurses, researchers, dieticians, LTC residents, family members, and representatives from associations including Osteoporosis Canada, LTC associations, an electronic medical record vendor, and family councils in LTC (Table 1). The meeting took place in October 2019 in a hotel meeting room. At the meeting, participants were divided into six focus groups and assigned seating to ensure equal representation of different stakeholders in each group. Focus group discussions lasted approximately 50 min in total and each group had one facilitator from the research team. Focus groups were guided by a semi-structured interview guide (Additional File 2) developed to elucidate implementation strategies and APEASE criteria evaluation to achieve the desired outcome behaviour — using the Fracture Risk CAP in routine clinical practice to identify residents at high risk for fractures and reduce the risk using clinical interventions.

### BCW Stage 1 - Understanding the behaviour

 Prior to the meeting, the research team conducted a systematic review which identified barriers and facilitators to implementing evidence-based guidelines in LTC [31]. The barriers and facilitators identified from the systematic review were mapped on to COM-B components (e.g. social opportunity) of the BCW.

At the meeting, we first presented information about fractures in LTC, the Fracture Risk CAP, knowledge translation, and the BCW and how to use it to the participants. The results of the previously completed systematic review and COM-B mapping were also presented to the participants. Next, participants were asked to discuss any additional barriers and facilitators they identified from their experiences that were not in our systematic review.

### BCW Stage 2 and 3 – Identify intervention options and Identify content and implementation options

Each focus group was assigned one of the six most frequently identified barriers from our systematic review (acceptance of guidelines, knowledge gaps, time constraints and inadequate staffing, reluctance to change, competing priorities, and compromised communication and information flow) and given a hand-out guide which included a description of the barrier, the linked COM-B component, and associated intervention functions (Additional File 3).

Participants were asked to identify specific implementation strategies that correspond to broad BCW intervention functions from their experience that could be used to help implement the Fracture Risk CAP. Participants were then asked to discuss whether the strategies they identified met the APEASE criteria. Participants were not asked explicitly to discuss modes of delivery, but the topic came up naturally as they discussed strategies and the APEASE criteria. Forming the focus groups around the identified barriers allowed for the participants to discuss how to overcome the specific barrier in multiple ways.

### Data collection and analysis

All focus group discussions were audio-recorded and transcribed verbatim by YB. For BCW Stage 3, each focus group facilitator also filled out notes on the barrier-to-intervention handouts given to each table. The data were analyzed using thematic content analysis [32, 33]. First, two team members (YB and CM) read the transcripts thoroughly to become familiar with the content of the transcripts. Next, the same team members independently and inductively identified relevant codes and condensed them into potential themes based on patterns of meaning. Subsequently, YB and CM reviewed the initial themes generated by checking them against the dataset and deliberated on discrepancies to split, combine, or discard them until consensus was reached. Finally, YB and CM redefined and named the themes. For BCW stage 1, barriers and facilitators themes were identified and presented with illustrative quotes. For BCW stage 2, descriptions of strategies detailed by the participants were grouped into themes and presented by the intervention function with which they were associated according to the BCW guide [30, 33]. We also present modes of delivery associated with the strategies and discussion points for the APEASE evaluation for BCW stage 3. This project was approved by the Hamilton Integrated Research Ethics Board.

## Results

Of the 47 experts we invited, 32 accepted our invitation, attended the meeting, and participated in focus group discussions (Table 1). Each focus group consisted of 5 to 6 participants. The remaining 15 invitees did not come due to scheduling conflicts with the one-day meeting.

**Table 1:** Participant characteristics

Characteristics	*N* = 32 (%)
***Health Professionals***	
Physician	10 (31.3)
Pharmacist	3 (9.4)
Physiotherapist	2 (6.3)
Kinesiologist	1 (3.1)
Nurse	2 (6.3)
Researcher	2 (6.3)
Dietician	3 (9.4)
***Residents and Family Members***	
Long-term care resident	1 (3.1)
Family member	1 (3.1)
***Representatives from Affiliated Organizations***	
Osteoporosis Canada	2 (6.3)
Long-term care associations	2 (6.3)
Electronic medical record vendor	1 (3.1)
Ontario Association of Residents’ Councils	1 (3.1)
Family Councils Ontario	1 (3.1)

### Barriers and facilitators to implementing the Fracture Risk CAP

Focus group participants identified several barriers and facilitators in the context of fracture prevention in LTC (Table 2). Specifically, barriers included language barriers between staff and residents or family members, lack of access to the required software, lack of expertise in the use of technology, training to a limited population. In particular, poor integration of physicians in the LTC care team was mentioned by multiple focus groups. Additional facilitators identified include having multiple checkpoints or another time to carry out an intervention if it was missed, sharing best practices across the LTC sector, technology, value propositions, and existing avenues to implement changes in LTC homes.

**Table 2:** Barriers and facilitators specific to fracture prevention

**Barrier**	Supporting Quotation
Language barrier between staff and residents/family	“Perhaps language barriers? […] knowing the complexity and the range of residents and families whereas staff may not speak the language.”
Lack of access to required software	“So in my home, we don’t use PointClickeCare [software]. So lack of access to the actual software or information systems.”
Lack of expertise in the use of technology	“Sometimes [technology] can also be a barrier.”
	“It can. Yeah. If it's not implemented correctly. It also depends on the level on the level of expertise with technology that the staff has.”
Training to a limited population	“We could do a lot of work to put together the CAP, and we do a lot of training to a very small proportion of people on what those CAPs mean, but the individuals that are really implementing theses at a clinical level, they never see these values.”
Lack of physician involvement	“Any time physicians are involved and are a key player in an intervention, they are not…they don’t have the same vested interest because they’re not really involved in quality improvement in our villages, because they have their own separate private practice.”
**Facilitator**	
Checkpoints – another time to carry out the intervention if it was missed due to time constraints	“Maybe they also need more than one point of care. So, if it’s missed because of the lack of time and resources. You never know what’s happening in a day. There should be another checkpoint.”
Sharing best practices across the care home sector	“A system that allows your support homes to share best and leading practices with other homes. As an alternative to compliance inspectors, facilitators of knowledge exchange and translation across the sector.”
Technology	“...definitely technology. Some technology that can help facilitate implementation.”
Value proposition	“Every time I knew there was something they were going to be losing, I try to find, what are they gaining? So we have a conversation here, can't do this for us, but you can do this for us.”
Existing avenues to implement change in LTCs	“I think a facilitator is that long-term care is really used to changing and there are a lot of the existing avenues that you can use to implement change, like best practice spotlights, all of the medication reduction that we've done related to antipsychotic use…residents and family councils, like there are huge number of avenues that you can access if you're trying to make a change and we're really used to change.”

### Strategies, modes of delivery, and APEASE

A summary of BCW intervention functions, strategies in the context of implementing the Fracture Risk CAP in LTC, their associated modes of delivery, and the APEASE criteria discussed in the focus groups is provided in Table 3.

Within the *training* function of the BCW, participants suggested mandatory training during onboarding and a propagative strategy where early change adopters are trained to train other staff on the usage of the Fracture Risk CAP. The participants believed that the strategy met the APEASE criteria as long as it does not shift the focus away from other tasks such as skin assessments for pressure ulcer prevention. Various strategies were identified in the *environmental restructuring* function. In particular, participants suggested workplace culture changes that promote teamwork among interdisciplinary staff and having consistent staffing, while cautioning that there may be risk of individuals performing tasks for which they were not trained. Another theme that many focus groups discussed was resident-centred care, although participants were concerned that lack of interest and availability from family may hinder the practicability, effectiveness, and acceptability of strategies in this theme. One focus group strongly emphasized integration of the Fracture Risk CAP into existing processes, which serves to minimize any additional workload placed on the staff and which they believed met all of the APEASE criteria.

Under *enablement*, the focus groups suggested developing software features that would facilitate the use of the Fracture Risk CAP. However, participants also pointed to limitations where developing software features may not be practicable due to development time and a side effect of taking the focus away from other important health conditions. *Modeling* was discussed in terms of building it into training using case studies and role-playing. For example, training sessions that incorporate multiple disciplines at the training stage could model the desired interdisciplinary collaboration in LTC homes. Moreover, *education* was grouped into education for staff and education for residents and their families. Discussions regarding education for staff centered around tailoring to specific roles, patient focused education, and taking an interprofessional approach with emphasis on physician involvement. Micro-learning and e-learning were also suggested to reduce costs and fatigue. On the other hand, education for residents and families was described both in terms of the importance of guidelines and how to advocate for their use, to be delivered through a pamphlet with actionable items and in-person during resident-family conferences. Participants believed that education should be targeted to residents and families of residents with high fracture risk, available in multiple formats, and easy to understand.

Within *persuasion*, participants advised to highlight stories that show the impact of fractures and the benefit of following the Fracture Risk CAP. Highlighting stories could be achieved using various modes of delivery and met the APEASE criteria as long as any visuals used are made for the homes, exhibit racial and gender diversity, and are available in multi-media formats. *Incentivization* was discussed in the form of social and material rewards for LTC homes with decreased fracture rates. Participants also critiqued the current funding model in Ontario where more funding is allocated based on the severity of the residents’ conditions in the LTC home, as they believed that it decreases the incentive for functional improvement. Furthermore, public benchmarking was proposed as a way of *coercion* by two focus groups, although one group pointed to a potential side effect, underreporting of cases of fractures. Finally, participants suggested some regulations that may be put in place as a form of *restriction*, which were ensuring that there is a care plan associated with an FRS score as a criterion for prescribing osteoporosis medication and changing policy to mandate use of up-to-date Fracture Risk Assessment and Prevention Tools.

Based on analysis of input from the stakeholders, we recommend incorporating strategies centred around the four themes that met the APEASE criteria (Table 4). We elaborate on these themes in the discussion.

**Table 3:** Potential strategies, mode of delivery, and APEASE criteria

BCWIntervention Function	Themes	Strategies in the context of implementing the Fracture Risk CAP	Mode of Delivery	Does it meet APEASE?
Training	Training on CAP usage	• Mandatory onboarding training	Online or in-person	Yes, with minor concern of shifting the focus from other health concerns in LTC (e.g. pressure ulcers)
		• Train the trainer – with follow-up support with multiple touchpoints, feedback, and regular positive reinforcement		
		• Train the early change adopters to be trainers		
Environmental restructuring	Culture change	• Change the social context by empowering personal support workers, volunteers, and families	LTC home policy and practices change	May not be acceptable to some due to sharing of power. Potential side effect of individuals performing tasks out of their scope.
		• Changing model of care to better include physicians and pharmacists		
		• Consistent staffing		
		• Promote team responsibility and promote inclusivity		
	Resident-centered care	• Sharing the CAP or assessment results with residents and families	Electronic portal. Resident-family conferences.	Family sharing portal may be costly to develop. May not be practicable, effective, or acceptable due to cost and lack of interest or availability for some families.
		• Family and resident-led huddles with staff during quarterly and annual reviews		
		• Working with family to balance individual rights, autonomy, freedom, and safety		
	Physical restructuring	• Physical modifications to the LTC home to reduce fractures and promote collaboration (e.g. handrails, open space)	Physical changes to the layout of the LTC home	Not discussed
		• Making the CAP easily accessible to all members on the care team	Virtual dashboard	Not discussed
	Minimize any increase in workload	• Standardized process once a high Fracture Risk Scale score is generated • Integration of the CAP into the existing processes (e.g. annual care conferences)	Care processes	Yes
Enablement	Software features	• Software add-on with the following features: flags staff only when meaningful changes occur, provide easy access to historical data, identify actionable things, perhaps targeted to person who can implement	Software	Development time may not be practicable. Side-effect: alarm fatigue taking away attention from other health conditions.
Modeling	Building modeling into training	• Case-study• Build into education by role-playing• Collaboration with multiple disciplines during the training stage to model real-life	In-person,videos	Not discussed.
Education	Education for staff	• Resources & materials tailored to different roles, easy access to reference tool that can be taken to bedside and used as part of training e.g., on tablet, on website • Patient-focused education for staff with simple and clear messaging • Take an interprofessional approach and improve physician involvement	Micro-learning and E-learning sessions,annual in-service,professional advisory meetings	More affordable if it’s online. Potential side effect of people burning out from training and time taken away from staff.
	Education for residents and families	• Importance of guidelines and how to advocate for following guidelines	One-page pamphlet with actionable items, resident-family conferences	Yes, as long as it is targeted to those at high fracture risk, available in multiple formats, factually accurate, and easy to understand.
Persuasion	Persuasion through stories	• Highlighting patient stories / identifying an important problem to show the impact of fractures • Value proposition by storytelling • Highlight reduced workload and increased QoL related to guideline usage	Posters or videos, social media and announcement channel or screen in LTC homes	Yes, as long as the visuals are made for the home, include gender and racial diversity, available in multi-media formats, and changed regularly
Incentivization	Social reward	• Recognition from organizations (i.e. Osteoporosis Canada)	Acknowledgement from organization – hard (i.e., plaque) or soft (i.e., seal of approval on website) copy	Not discussed
	Material reward	• Award the ward with the lowest fracture rate • Flip case-mix funding to incentivize functional improvement, since the current case-mix model decreases that incentive	Financial, food (e.g., pizza party), material good (e.g., t-shirts)	Not discussed
Coercion	Public benchmarking	• Public benchmarking, against Canadian provinces or other countries	Publicly accessible online dashboard	Potential side effect of underreported cases.
Restriction	Regulations	• Fracture Risk Scale score and associated care plan as criteria for use of osteoporosis medication • Ministry of Health mandates use of up-to-date Fracture Risk Assessment and Prevention Tools	Policies and procedures	Not discussed

*LTC* Long term care; *CAP* Clinical assessment protocol; *BCW* Behaviour Change Wheel

**Table 4:** Recommended themes of strategies to be incorporated into an implementation intervention for the Fracture Risk CAP

Recommendations	Description	COM-B Linkage
Minimize any increase in workload	Use standardized processes and integrate the Fracture Risk CAP into existing LTC processes.	Capability and opportunity to overcome time constraints and inadequate staffing and reduce competing priorities.
Training on CAP usage	Training local trainers on how to use the Fracture Risk CAP during mandatory onboard training.	Capability and opportunity to overcome knowledge gaps and improve information flow.
Education for residents and families	Educating residents and families on the importance of guidelines and how to advocate for following guidelines.	Motivation and social opportunity to overcome unacceptance of guidelines and reluctance to change.
Persuasion through stories	Highlighting patient and staff narratives to show the impact of fractures and the value of using the Fracture Risk CAP	Motivation and social opportunity to overcome unacceptance of guidelines and reluctance to change.

## Discussion

 This study used the Behaviour Change Wheel, informed by stakeholder interviews, to identify strategies for addressing common barriers (unacceptance of guidelines, knowledge gaps, time constraints and inadequate staffing, reluctance to change, competing priorities, and compromised communication and information flow) to implementing evidence-based guidelines in LTC identified through systematic review of the literature and in the context of fracture risk prevention.

In addition to the aforementioned barriers, our interview participants also noted other barriers and facilitators from their experiences. These included language barriers, lack of access to required software, lack of expertise in the use of technology, training for a limited population, and poor integration of physicians on the LTC care team. While the literature on knowledge translation in the LTC setting rarely mention language barriers as an impediment to evidence uptake, language barriers have been demonstrated to adversely affect the access and quality of health care in Canada and the United States [34, 35]. Indeed, residents who are not proficient in the language spoken by the LTC care team face additional communication difficulties that may impede their care [36]. Therefore, strategies to overcome language barriers should be considered when designing implementation interventions if applicable to the participating LTC home. Furthermore, several LTC stakeholders in our study discussed how the poor integration of physicians in the LTC care team may affect fracture prevention. When physicians need to travel between several LTC homes, health conditions such as fracture risk that are often placed at low priority compared to more acute conditions such as heart diseases are sometimes overlooked despite their severe consequences [37, 38]. However, evidence suggest that involving physicians on the care team could reduce medication costs and hospital transfers [39, 40]. Therefore, the incorporation of physicians on the LTC care team requires further attention.

Facilitators discussed by our participants include checkpoints and having another time to carry out an intervention, sharing best practices across the LTC sector, technology, value propositions, and existing avenues to implement changes in LTC homes. In particular, when discussing existing avenues to change in LTC homes, a participant noted “being used to change” as a facilitator to evidence uptake and pointed to previous work done on antipsychotic use reduction in LTC that may pave the way for implementing the Fracture Risk CAP [41]. On the other hand, studies on implementation of evidence-based tools and guidelines have noted change fatigue as a barrier to knowledge translation [42, 43]. Therefore, even if LTC homes have established avenues for change, it’s important to avoid change fatigue by considering the LTC staff’s attitudes on and capacity for new innovations.

Implementation strategies identified by the LTC stakeholders were grouped into 14 strategy themes, four of which met the APEASE criteria: minimizing any increase in workload, training on CAP usage, education for residents and families, and persuasion through stories. Other strategies in Table 3 may also be considered for intervention implementation depending on the context of the LTC home (i.e. availability of resources). Many of the strategy themes identified are likely important when implementing other guidelines or tools in the LTC setting though the specific strategies were discussed in the context of implementing the Fracture Risk CAP. For example, strategies that address education and include modeling are likely needed across many guidelines. Nevertheless, we suggest future researchers interested in knowledge translation identify unique barriers and facilitators for their guideline topic in addition to general barriers and consider a theory-informed approach to developing implementation interventions.

According to the BCW guide, there must be the capability (i.e. knowledge, skills, physical abilities), opportunity (i.e. a conducive social and physical environment), and motivation for any behaviour to occur [30]. Frequently reported barriers across many guideline topics include lack of time and lack of staff, which interferes with the LTC staff’s capability and opportunity to adhere to the guideline [44].A strategy theme that improve capability and opportunity of LTC staff, was strongly recommended by the participants, and met the APEASE criteria is minimizing any workload increase associated with the guidelines as part of the intervention function, *environmental restructuring*. Since staffing and workload is often a concern in the LTC sector, implementing new guidelines in LTC homes should not overburden the staff. Using standardized processes in LTC to simplify and decrease the workload has been recommended in the literature [45]. However, exclusive institutional reliance on standardized processes may fail to achieve individualized care planning, so residents’ preferences and input by care aides should be considered [46]. Leveraging existing processes in the LTC homes may also be a way to minimize workload. Examples of processes in LTC homes that could be leveraged for implementing the Fracture Risk CAP include discussing its usage during annual care conferences and adding it to the Exercise and Falls Prevention Program in Ontario [47].

Second, training on CAP usage under the intervention function, *training*, was recommended to increase capability and opportunity of LTC staff to use the Fracture Risk CAP. Participants suggested including the training in the mandatory onboard training for all LTC staff and using the “train-the-trainer” model as a way to disseminate additional knowledge and skills needed to use the tool. The “train-the-trainer” model is a framework for training internal employees to train others in the organization [48]. This model was reported to better embed knowledge and enhance sustainability of evidence uptake due to benefits including having local trainers who learn better because they need to teach others and who can tailor the training to the context of the organization [48].

The third recommendation that met the APEASE criteria is “education for residents and families” under the intervention function *Education.* In two qualitative studies examining the experience of care staff in implementing evidence-based guidelines, involving families and residents when introducing evidence-based interventions were perceived by care staff as a facilitator to implementation [49, 50]. Educating residents and families on not only the importance of fracture prevention but also how to advocate for the use of evidence-based interventions may increase the motivation and opportunity for the staff to carry out the guideline.

Lastly, the fourth recommendation that met the APEASE criteria is persuasion through stories under the intervention function *persuasion* to increase LTC staff’s motivation and social opportunity to use the Fracture Risk CAP. Focus group participants proposed highlighting patient and staff narratives to show the impact of fractures and the value of using the Fracture Risk CAP. A narrative review in using persuasive interventions to optimize antimicrobial use in healthcare found that taking a multi-modal approach and involving all stakeholders such as patients and families were associated with successful knowledge translation[51]. Furthermore, research in psychology found that embedding facts within a story is more effective for persuasion that facts alone [52]. Persuasive strategies have not been formally studied for implementing fracture prevention interventions in LTC and may be an interesting area for future work.

Despite not fully meeting the APEASE criteria, other implementation strategies identified in this study may be considered when designing an implementation intervention in LTC. For example, several strategies discussed by multiple focus groups fall under the theme “culture change” in *environmental restructuring*, which encompasses changing the social context to empower personal support workers (or care aides), volunteers, and families, integrating physicians and pharmacists in the care team, having consistent staffing, and promoting team responsibility. Care aides are workers who have the most direct contact with LTC residents, but also those who have the “least training, authority, and status within the system” [53]. Interestingly, empowering care aides and ensuring consistent staffing can positively influence resident outcomes including lower pressure ulcer incidence rates and higher social engagement scores [54]. Similarly, empowerment of nursing assistants improved service quality more than the empowerment of nurses [55], which demonstrates the importance of focusing on this role in LTC.

A strength of our study is the use of a comprehensive framework to guide the design and analysis of implementation strategies. Our behaviour change theory-informed approach allowed us to generate a systematic understanding of implementation problems and the generation of strategies to overcome those problems. Thus, the recommended strategies are more likely to be successful. Another strength is the wide range of roles in LTC that we consulted to identify and evaluate the feasibility of the strategies, which offers many different perspectives for how to best implement the Fracture Risk CAP. However, as recruitment was performed through word-of-mouth and participants were known to the researchers, our findings may be biased towards LTC stakeholders who value fracture prevention and have views similar to ours. Additionally, we only recruited a small portion of LTC residents and relatives, which limited input from these perspectives in several focus groups. Due to time constraints, our focus group participants did not fully discuss the APEASE evaluation for all the strategies. Even though the participants were asked to use APEASE to evaluate the strategies they favoured most, the strategies that were not evaluated may potentially also fit the criteria. Moreover, there is subjectivity in how we encoded the strategies into different themes and intervention functions as there are several possible ways to link strategy themes to intervention functions that depend on interpretation of transcripts. Furthermore, data saturation appeared to have been reached as many themes came up across different focus groups, but we cannot be certain because all the focus groups were conducted in the one-day national meeting. While we evaluated the feasibility of most implementation strategies using the APEASE criteria in consultation with stakeholders, we could not conclude which ones are most effective. Our next steps are to prioritize and examine the effectiveness of these strategies to change practice and influence resident outcomes as we implement the Fracture Risk CAP.

## Conclusions and implications

 Guided by the BCW and in consultation with LTC stakeholders, we identified several strategies that are feasible to overcome anticipated barriers to implementing evidence-based guidelines in LTC homes.

Based on the APEASE criteria, we recommend strategies centred around minimizing any increase in workload, training on CAP usage, education for residents and families, and persuasion through stories. Other themes of strategies that may be considered are culture change, resident-centred care, physical restructuring, software features, modeling in training, education for staff, social rewards, material rewards, public benchmarking, and regulations. Results from this work will be crucial to increase usage of the Fracture Risk CAP and consequently improve identification and management of LTC residents at high fracture risk.

## Supplementary Information

**Additional file 1**

**Additional file 2**

**Additional file 3**

## Data Availability

The datasets generated and/or analysed during the current study are not publicly available due to limits placed on who has access to data during the informed consent process but are available from the corresponding author on reasonable request.

## Abbreviations

LTC: Long-term care
CAP: Clinical assessment protocol
BCW: Behaviour Change Wheel
COM-B: Capability, opportunity, motivation, behaviour
APEASE: Affordability, Practicability, Effectiveness, Acceptability, Safety, Equity
