# Supplementary material for: Strategies for the implementation of an electronic fracture risk assessment tool in long term care: a qualitative study
Source: BMC Geriatr. 2021 Aug 21;21:467. doi: 10.1186/s12877-021-02388-3 (PMC8379826; doi:10.1186/s12877-021-02388-3)
Supplement: Supplementary file 1 — Additional file 1 [file 12877_2021_2388_MOESM1_ESM.pdf]

Supplementary Table 1. Definitions of the intervention functions provided by the Behaviour Change Wheel[1]

	Definition	Example
Education	Increasing knowledge or understanding	Providing information to promote healthy eating
Persuasion	Using communication to induce positive or negative feeling or stimulate action	Using imagery to motivate physical activity
Incentivisation	Creating an experience of reward	Using prize draws to induce attempts to stop smoking
Coercion	Creating an expectation of punishment or cost	Raising the financial cost to reduce smoking
Training	Imparting skills	Driver training to increase safe driving
Restriction	Using rules to reduce the opportunity to engage in the target behaviour or competing behaviours	Prohibiting sales of alcohol to people under 18
Environmental Restructuring	Changing the physical or social context	Providing on-screen prompts for nurses to monitor fall risk
Modeling	Providing an example for people to aspire to or imitate	Using TV drama scenes involving safe-sex to increase condom use
Enablement	Increasing means/reducing barriers to increase capability or opportunity (beyond education, training, environmental restructuring)	Surgery to reduce obesity, prostheses to promote exercise, behavioural support for smoking

Supplementary Table 2. APEASE definitions provided by the Behaviour Change Wheel[1]

Affordability	The intervention can be given to, or accessed by, all those for whom it would be relevant or of delivered benefit to within an acceptable budget
Practicality	The intervention can be delivered as designed. E.g. An intervention may only be useful when delivered by highly trained staff, which may not be achievable
Effectiveness/ Cost-effectiveness	The effect size of the intervention relative to the desired objectives in a real-world context (public, professional, political)
Acceptability	The extent to which an intervention is judged to be appropriate by relevant stakeholders
Side effects/ Safety	The unintended consequences of an intervention
Equity	The extent to which an intervention may increase or decrease disparities in standard of living, wellbeing or health between different sectors

1. Michie S, van Stralen MM, West R. The behaviour change wheel: A new method for characterising and designing behaviour change interventions. Implement Sci. 2011;6(1):42. doi:10.1186/1748-5908-6-42
