# Supplementary material for: Strategies for the implementation of an electronic fracture risk assessment tool in long term care: a qualitative study
Source: BMC Geriatr. 2021 Aug 21;21:467. doi: 10.1186/s12877-021-02388-3 (PMC8379826; doi:10.1186/s12877-021-02388-3)
Supplement: Supplementary file 2 — Additional file 2 [file 12877_2021_2388_MOESM2_ESM.pdf]

Preventing Fractures in Home and Long-term Care Meeting

Wednesday, October 16th, 2019

ALT Hotel Toronto Airport

7:30am-4:00pm

GENERAL NOTES

- Each participant has been assigned to a certain table (including you!)
- There will be an audio-recorder placed at each table
- For each interactive session, audio-record the conversation at the table
    - Tell the participants you are going to start recording, if they wish to say something they don't want recorded they can tell you and you can pause the recording, then restart it once they are done
    - At the beginning of each recording – start the recording by identifying the table you are at (E.g., red, blue) and which interactive activity it is (e.g., 1, 2).
- Arrive for the day 7:30am, we will be done at 4:00pm

INTERACTIVE ACTIVITY 1

Purpose: To identify goals and outcomes of implementing the Fracture Risk CAP that can be used in our larger grant application

Time: 9:45-10:15am

Location: Round tables and on the wall

Set-Up/Materials: Post-it notes, audio-recorder

Task:

- Participants are asked to identify their top 3 goals and outcomes of implementing the Fracture Risk CAP
- They will be provided with a definition of goals and outcomes
- Participants write their top three goals on the pink post-it notes, and their top three outcomes on the green post-it notes
- Participants go place their three goals and outcomes under the goals and outcomes sheets placed at the side of the room – they put their number one choice under the 1, second under the 2, and third under the 3.

Your role:

- Audio-record any conversation at the table
- Provide clarification of the task

- Assist any participants who need help placing the post-its up on the wall (some family and resident participants are attending)

INTERACTIVE ACTIVITY 2

Purpose: To help us identify realistic knowledge translation interventions that can be used to help implement the fracture risk CAP for our grant application

Time: 10:50-12:00

Location: Round tables

Set-Up/Materials: Audio-recorder, barriers/facilitators handout

Task 1 (3 minutes):

- Participants are asked if there are any other barriers or facilitators that we have not identified in the systematic review

Your role:

- Audio-record the conversation
- Ask participants if there are any other barriers or facilitators that they can see to implementing the fracture risk CAP that we have not identified
    - Probing questions:
        - Do you see any other barriers to implementing guidelines in your facility/home?
        - Would anything else make it harder or easier to put this into practice?

Task 2 – Step 1 (20 minutes):

- Complete step 1 of the handout – participants are to identify examples of these intervention functions, e.g., real world examples of what they mean. For example, for training what could that training actually look like?

Your role:

- Audio-record the conversation
- Use the reference guide on your table to go over the definition of the intervention function
- Ask participants to brainstorm what that might look like in the real world
    - Probing questions:
        - What might that look like in practice or the real world?
        - How could you do that (e.g., training, education, enablement) in practice?
        - What are some ideas for this type of intervention?

- Record their answers on the handout

Task 2 – Step 2 (20 minutes):

- Complete step 2 of the handout – identify if the interventions they have identified are realistic to implement, and why or why not.

Your role:

- Audio-record the conversation
- Ask participants if their identified intervention functions meet the APEASE criteria?
- Use the reference guide on your table to review the definition of the APEASE criteria
- For each intervention function (e.g., training, restriction) identify whether it meets the APEASE criteria, record reasons why or why not
    - Probing questions:
        - Is this intervention (e.g., training) affordable/practicable/acceptable/have side effects/safety issues/equitable?
        - Why or why not?
