# Supplementary material for: Strategies for the implementation of an electronic fracture risk assessment tool in long term care: a qualitative study
Source: BMC Geriatr. 2021 Aug 21;21:467. doi: 10.1186/s12877-021-02388-3 (PMC8379826; doi:10.1186/s12877-021-02388-3)
Supplement: Supplementary file 3 — Additional file 3 [file 12877_2021_2388_MOESM3_ESM.pdf]

Barrier: Competing Priorities

Staff burdened with too many tasks to place guideline adherence at high priority

COM-B Element: Social Opportunity

Interventions

STEP 1

Modelling	Restriction	Environmental Restructuring	Enablement

APEASE

STEP 2

Modelling	Restriction	Environmental Restructuring	Enablement
Affordability ◯	Affordability ◯	Affordability ◯	Affordability ◯
Practicality ◯	Practicality ◯	Practicality ◯	Practicality ◯
Effectiveness/ Cost-effectiveness ◯	Effectiveness/ Cost-effectiveness ◯	Effectiveness/ Cost-effectiveness ◯	Effectiveness/ Cost-effectiveness ◯
Acceptability ◯	Acceptability ◯	Acceptability ◯	Acceptability ◯
Side effects/ Safety ◯	Side effects/ Safety ◯	Side effects/ Safety ◯	Side effects/ Safety ◯
Equity ◯	Equity ◯	Equity ◯	Equity ◯

Why or why not?	Why or why not?	Why or why not?	Why or why not?
